# Ceramide Metabolism and Parkinson’s Disease—Therapeutic Targets

**DOI:** 10.3390/biom11070945

**Published:** 2021-06-25

**Authors:** Antía Custodia, Marta Aramburu-Núñez, Clara Correa-Paz, Adrián Posado-Fernández, Ana Gómez-Larrauri, José Castillo, Antonio Gómez-Muñoz, Tomás Sobrino, Alberto Ouro

**Affiliations:** 1 Clinical Neurosciences Research Laboratories, Health Research Institute of Santiago de Compostela (IDIS), Travesa da Choupana s/n, 15706 Santiago de Compostela, Spain; antia.custodia.malvido@sergas.es (A.C.); marta.aramburu.nunez@sergas.es (M.A.-N.); clara.correa.paz@sergas.es (C.C.-P.); adrian.posado.fernandez@sergas.es (A.P.-F.); jose.castillo.sanchez@sergas.es (J.C.); 2 Department of Biochemistry and Molecular Biology, Faculty of Science and Technology, University of the Basque Country, P.O. Box 644, 48980 Bilbao, Spain; ana.gomezlarrauri@osakidetza.eus (A.G.-L.); antonio.gomez@ehu.eus (A.G.-M.); 3 Respiratory Department, Cruces University Hospital, Barakaldo, 48903 Bizkaia, Spain

**Keywords:** ceramide, sphingolipids, Parkinson’s disease, neurodegeneration, sphingomyelinase, ceramide synthase, β-GCase, sphingolipidomics

## Abstract

Ceramide is a bioactive sphingolipid involved in numerous cellular processes. In addition to being the precursor of complex sphingolipids, ceramides can act as second messengers, especially when they are generated at the plasma membrane of cells. Its metabolic dysfunction may lead to or be a consequence of an underlying disease. Recent reports on transcriptomics and electrospray ionization mass spectrometry analysis have demonstrated the variation of specific levels of sphingolipids and enzymes involved in their metabolism in different neurodegenerative diseases. In the present review, we highlight the most relevant discoveries related to ceramide and neurodegeneration, with a special focus on Parkinson’s disease.

## 1. Introduction

Parkinson’s Disease (PD) is the second most common neurodegenerative disease [1]. PD affects 1% of the population over 60 years of age, with a higher risk of developing the disease in males [2,3]. The annual economic burden of PD in the European healthcare system per patient ranges from €2600 to €10,000 [4]. The disease presents with symptoms of motor impairment such as bradykinesia, rigidity, tremor, postural instability, and difficulty in speaking and swallowing. As for non-motor symptoms, patients present sleep disturbance, depression, cognitive impairment, sensory abnormalities, or autonomic dysfunction [1,2].

PD is characterized by the accumulation of misfolded α-synuclein (α-syn) in inclusions called Lewy bodies located in the substantia nigra of the central nervous system, resulting in the loss of dopaminergic neurons in substantia nigra pars compacta and striatal dopamine, which are responsible for the motor symptoms of the disease. Lewy bodies have also been found in other areas of the brain such as raphe nuclei, locus coeruleus, brainstem reticular formation, the dorsal motor nucleus of the vagus nerve, amygdala, hippocampus and nucleus basalis of Meynert, to which non-motor symptomatology is attributed [1,2].

As for the mechanisms responsible for PD, neuronal death and neurodegeneration have been linked to oxidative stress, vascular disfunction, tumor progression, altered mitochondrial, autophagy and proteolysis functions, inflammation, excitotoxicity and lysosomal storage disorders (LSD) [2,5]. In addition, it was reported that alterations in sphingolipid metabolism in the early stages of the disease may be linked to an increased risk of developing PD with dementia, while regulating the levels of certain sphingolipids by enzymatic regulation can slow the development of the disease [6].

Sphingolipids are widely distributed in the organism, including the central and peripheral nervous system [7,8]. The closest association of sphingolipids with neurodegenerative diseases was related to their structural function, especially the glycosphingolipids, as the main component of the plasma membrane of oligodendrocytes and myelin [9]. However, several studies have demonstrated the implication of sphingolipids in several key biological processes such as cellular proliferation and migration, differentiation, autophagy, apoptosis, senescence, and inflammation [8,10,11,12,13,14]. Recent works on transcriptomics and electrospray ionization mass spectrometry analysis (sphingolipidomics) have demonstrated the variation of specific levels of sphingolipids and enzymes involved in their metabolism in different neurodegenerative diseases [15,16,17].

Ceramides (Cers) have a dual role in cell biology since they are precursors of complex sphingolipids and second messengers to regulate cell homeostasis [7,8]. Ceramide (Cer) is highly expressed in neurons and modulates neuronal signaling, synaptic transmission, cell metabolism, neuron-glia interaction and cell survival [18,19,20]. The intracellular accumulation of Cer has been noticed as a critical step to neurodegeneration [21]. Neurodegenerative diseases and aging have a direct connection with oxidative stress. The increase in oxidative stress induces stimulation of the sphingomyelinase (SMase) activity and the consequent elevation of intracellular Cer concentration in neurons and oligodendrocytes [21,22]. Furthermore, mutations in different enzymes involved in the metabolism of Cer have been implicated in the development of neurodegenerative diseases. It should be noted that part of lipid metabolism takes place in lysosomal compartments. Thus, it is important to discriminate between lysosomal enzyme alterations, which would be part of LSD and non-lysosomal enzymes [23]. In this review, we will discuss the involvement of sphingolipids in neurodegeneration with special emphasis on PD. 

## 2. Sphingolipid Metabolism

**Figure 1:**
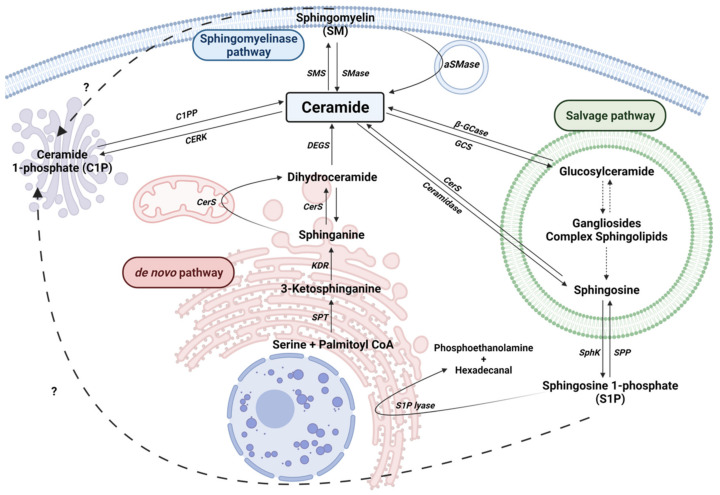
Sphingolipid metabolism. Solid arrows represent single reactions, whereas dashed arrows represent various step reactions. Interrogation marks with dashed arrows indicate unidentified mechanisms. Sphingomyelinase (SMase), sphingomyelin synthase (SMS), acid-sphingomyelinase (aSMase), Ceramide 1-phosphate phosphatase (C1PP), ceramide kinase (CERK), Serine palmitoyl transferase (SPT), 3-ketosphinganine reductase (KDR), Ceramide Synthase (CerS), sphingosine kinase (SphK), sphingosine 1-phosphate phosphatase (SPP), Sphingosine 1-phosphate lyase (S1P lyase) are represented by their acronyms.

Cers are considered the central hub of sphingolipid metabolism and can regulate key metabolic functions. In particular, Cers are potent inducers of cell cycle arrest and apoptotic cell death [13,24], and are implicated in inflammatory responses related to microbial infection, asthma, cardiovascular diseases or chronic obstructive pulmonary disease (COPD) [25,26]. Moreover, an unbalance of intracellular Cer levels can lead to neurological and neuroinflammatory diseases. Cers are synthesized mainly by three major pathways. Furthermore, Cer can be synthesized by the dephosphorylation of ceramide 1-phosphate (C1P) by ceramide 1-phosphate phosphatase (CPP). In addition to producing Cer, the metabolites of complex sphingolipid catabolism lead to the production of another bioactive molecules, such as sphingosine 1-phosphate (Figure 1). 

### 2.1. The de novo Pathway

This pathway takes place in the endoplasmic reticulum (ER) where serine palmitoyltransferase (SPT) catalyzes the condensation of palmitate and serine to form 3-ketosphinganine (also called 3-ketodehydrosphingosine). Recent structural studies on SPT revealed a symmetrical dimer protein anchored to the ER membrane by six α-helices. The complex is formed by a single molecule of SPTLC1, SPTLC2, ssSPTa/b (two small subunits that enhance enzyme activity and also specify acyl-CoA substrate) and four regulatory subunits, ORMDLs (homologs of the yeast and plant Orms) [27]. Then, 3-keto-dihydrosphingosine reductase (KDR) produces sphinganine. Ceramide synthase (CerS) can then catalyze the formation of dihydroceramide (dhCer) through the incorporation of acyl-CoA of different chain lengths to sphinganine. There are six different isoforms of CerS (CerS1-6) identified in mammals and plants [28]. Specifically, CerS1 is highly expressed in the nervous system and skeletal muscles, but is almost undetectable in other types of tissue. CerS1 mainly generates 18 carbon chain Cer (C_18_-Cer), whereas CerS2 produces C_22/24_-Cer, CerS3 produces C_26_-Cer, CerS4 generates C_18/20_-Cer, CerS5 synthesizes C_14/16_-Cer and CerS6 forms C_14/16_-Cer. Cers with different acyl chain lengths have been detected in the mitochondria of the brain [29,30]. Furthermore, in brain tissue, CerS1, CerS2 and CerS6 enzymes were localized to the inner and outer mitochondrial membrane, and can induce the synthesis of C_18_-Cer, C_22_-Cer and C_16_-Cer, respectively [31]. Interestingly, mitochondrial CerS was associated with mitochondrial injury in cerebral ischemia/reperfusion with increased production of Cer [31]. The last step of this pathway is catalyzed by a dihydroceramide desaturase (DEGS), which introduces a double bond in position 4-5 trans of dhCer. DEGS is localized to the cytosolic leaflet of the ER membrane. In particular, genetic manipulation of the *DEGS* gene by tissue-specific deletion reduced hepatic steatosis and attenuated insulin resistance [32]. *DEGS* polymorphisms have been associated with the develop of cognitive impairment in schizophrenia [33]. Recently, *DEGS* mutation has been described to produce hypomyelination and degeneration of both central and peripheral nervous systems [34].

### 2.2. The Sphingomyelinase (SMase) Pathway

SMases are enzymes that hydrolyze sphingomyelin (SM) at the plasma membrane of cells to generate Cer and phosphocholine. SM hydrolysis is considered a fast mechanism for the production of Cer. There are five different types of SMases [35], and these have been classified according to their ion dependence, location and optimal pH. These include lysosomal and plasma membrane acid SMase (aSMase) [36], endoplasmic reticulum/nucleus and plasma membrane neutral Mg^2+^-dependent and neutral Mg^2+^-independent SMase (nSMase) [37], alkaline SMase (alkSMase), which is present in the intestinal tract and human bile [35,38], and a Zn^2+^-dependent secreted form of aSMase [39]. Meanwhile, aSMase and nSMase are implicated in cellular signaling, whereas alkSMase is implicated in the degradation of SM incorporated in the diet. It should be noted that aSMase and nSMase increase their activity by the action of pro-inflammatory stimuli, such as Tumor Necrosis Factor α (TNF-α), Interleukin-1β (IL-1β) or cytosolic phospholipase A_2_ (cPLA_2_), and it leads to elevation of intracellular Cer concentrations [40,41,42,43]. In addition, these enzymes are activated by some anticancer drugs, and by irradiation of cells with ultraviolet (UV) or ionizing radiation [44]. Mutations in the aSMase (*SMPD1*) gene results in disfunction of cholesterol and lipids metabolism, leading to Niemann-Pick’s disease [45,46].

There are different nSMase isoforms that have been characterized under different experimental settings [47]. The nSMase1 isoform (*SPMD2* gene) is expressed in all cell types and highly enriched in the kidney. The nSMase2 isoform (*SPMD3* gene) has a different domain structure than nSMase1. Contrary to nSMase 2, nSMase1 has two transmembrane domains, and instead has one collagen-like domain and two hydrophobic domains [48]. Of interest, nSMase2 is highly expressed in brain tissue [49]. Lastly, nSMase3 (*SMPD4* gene) is ubiquitously expressed in all cell types. All of these SMase isoforms are Mg^2+^-dependent for expression of their activity. Dysregulation or stimulation of nSMase activity has been related to PD, Alzheimer’s disease, cognitive dysfunction or cerebral ischemia recovery [47,48,50,51,52].

### 2.3. The Salvage Pathway

This pathway involves a series of catabolic reactions that result in degradation of complex sphingolipids in acidic compartments, such as lysosomes. Complex sphingolipids, such as gangliosides (GM1, GM2 or GM3) or globosides, can be degraded to Lactosyl-ceramide (LacCer) by different reactions. Then, LacCer can be converted to Glucosyl-Ceramide (GlcCer) by LacCer hydrolase. Acid β-glucosidase 1 (β-GCase), encoded by GBA1 gene, converts GlcCer to lysosomal Cer. Deficiencies or dysfunction of this enzyme can lead to the accumulation of GlcCer and the development of the lysosomal storage disease known as Gaucher’s disease. Moreover, mutations in the *GBA1* gene with loss of function have been linked to PD [53,54]. Contrarily, glucosylceramide synthase (GCS) transforms Cer into GlcCer. Once generated, Cer can be converted to sphingosine (Sph) by ceramidases. These enzymes differ in their optimal pH. There are three alkaline ceramidases (ACER1, ACER2 and ACER3), an acidic ceramidase (ASAH1) and a neutral ceramidase (ASAH2) [55,56]. ASAH1 is ubiquitously expressed in lysosomal compartments while ASAH2 is localized in plasma membranes, and mainly expressed in the small intestine and colon [55]. Sph is released to the cytosol and transformed to Cer by the activity of CerS in ER.

### 2.4. Ceramide Kinase/Ceramide 1-Phosphate Phosphatase (CerK/CPP) and Sphingosine Kinase/Sphingosine 1-Phosphate Phosphatase (SphK/SPP) Axis

Another relevant enzyme is Ceramide kinase (CerK), which phosphorylates Cer to produce Ceramide 1-phosphate (C1P). C1P is a key regulator of cell proliferation, survival and inflammation. The signaling pathways involved in C1P actions include mitogen-activated protein kinase kinase (MEK)/extracellularly regulated kinases (ERKs) 1/2, the mammalian target of rapamycin (mTOR), phosphatidylinositol 3-kinase (PI3K)/Akt, or protein kinase C-α [57,58,59], c-Jun N-terminal kinase (JNK) [60], or stimulation of vascular endothelial cell growth factor (VEGF) secretion [61]. Additionally, C1P-promoted cell survival implicates upregulation of inducible nitric oxide synthase (iNOS) expression [62], direct inhibition of aSMase [63,64] or SPT [65] and activation of the PI3K/Akt pathway [66]. It should be noted that C1P is implicated in the regulation of autophagy [67].

Sph that is released from the lysosomes to the cytosol in the Salvage pathway can be phosphorylated by sphingosine kinase (SK) to form S1P. It is one of the most studied sphingolipids. S1P has been described as a potent regulator of inflammatory processes through its union with specific membrane receptors. Thus far, five S1P receptors (S1PR_1-5_) have been described [68]. It is worth highlighting its involvement in glia activation processes, ischemic stroke and inflammatory processes in the vascular endothelium [69,70,71].

Dephosphorylation of S1P to Sph is due to the activity of S1P phosphatase (SPP) or by lipid phosphate phosphatase (LPP) activity [13]. However, S1P can be catalyzed by S1P lyase to produce hexadecenal and phosphoethanolamine in ER [72]. Of interest, it was observed, in both in vitro and in vivo models with a lack of S1P lyase an accumulation of β-amyloid and α-syn, promoting dysfunction of neuronal autophagy. In addition, the treatment with phosphoethanolamine restored autophagy, decreasing the deposits of β-amyloid and α-syn [73].

## 3. Neurodegeneration and Sphingolipid Metabolism 

Alzheimer’s disease (AD) is the most prevalent neurodegenerative disease. It is characterized by extracellular deposits of β-amyloid (previously cleaved by secretases), called senile plaques, and intracellular build-up of hyperphosphorylated Tau protein in neurofibrillary tangles [74,75]. High levels of different species of Cer have been found in human samples from AD patients [76,77,78]. In addition, senile plaques were enriched in C_18:1/18:0_ and C_18:1/20:0_-Cer [79]. Likewise, aSMase and nSMase2 were found overexpressed in AD brain samples, correlated with increased Cer levels in blood [77]. Moreover, treatment with a cell-permeable analog of Cer (C_6_-Cer) or stimulation of endogenous Cer by nSMase activation stabilized β-site amyloid precursor protein cleaving enzyme 1 (BACE1) increasing β-amyloid accumulation [80]. Interestingly, β-amyloid has been reported to stimulate SMase activity in neurons [43,81,82], oligodendrocytes [83], dendritic [82] and endothelial cells [84], stimulating Cer accumulation and, thereby cell death. Additionally, overexpression of S1P lyase has been described to reduce β-amyloid production in N2a neuroblastoma cells [85]. Furthermore, it was observed a significant reduction of SphK1 and an increase of S1P lyase in AD human brain samples [86].

Different genetic diseases disrupt the metabolism of several molecules in the lysosomes, knowns as Lysosomal storage diseases (LSD). One of the main causes is lipid metabolism dysfunction, due to the alteration of enzymes such as aSMase or β-GCase [23,87,88,89]. LSD include different diseases, such as Niemann-Pick’s disease, Gaucher’s disease, Farber’s disease, Krabbe’s disease, Fabry’s disease, Tay-Sach’s disease, Sandhoff’s disease and ganglioside synthase deficiency. Lysosomal lipid storage occurs in all types of the disease, again highlighting the link between altered sphingolipid metabolism and neurodegeneration.

Niemann-Pick’s disease is a genetic disease that can be caused by two different types of mutations. Mutations in the *SMPD1* gene lead to build-up of SM and the develop of Niemann-Pick’s disease type A and B [45,90]. Meanwhile, mutations in NPC Intracellular Cholesterol Transporter 1 or 2 (*NPC1* or *NPC2*) alter cellular cholesterol trafficking and lipid metabolism disruption, leading to Niemann-Pick’s disease type C1 and C2 [91]. Recently, Torres et al. have shown that ASAH1 is downregulated in patients with Niemann-Pick’s disease type C1 [92]. They have also observed that the overexpression of ASAH1 improves mitochondrial function and reduces oxidative stress by decreasing STARD1.

Gaucher’s disease is due to a mutation in the gene encoding β-GCase (*GBA*), resulting in a deficit of the lysosomal enzyme, leading to an accumulation of GlcCer mainly in macrophages [53]. Elevated levels of glucosylsphingosine (GlcSph) were also found in the brain and were correlated with the phenotype of the disease [93]. Gaucher disease is associated with an increased risk of PD and dementia, since *GBA* deficiency increase α-syn aggregates [5].

Faber’s disease is caused by mutations in the *ASAH1* gene, leading to an accumulation of Cer and cerebral atrophy. Interestingly, a rare epileptic disorder known as spinal muscular atrophy with progressive myoclonic epilepsy (SMA-PME) is also associated with ASAH1 deficit [94]. Recently, C_26_-Cer was proposed as a biomarker for Faber’s disease diagnosis [95].

Krabbe’s disease is a genetic disease characterized by extensive demyelination, apoptosis of oligodendrocytes and Schwann cells and neurodegeneration due to mutations in the *GALC* gene that encode for galactocerebrosidase [96]. Recently, the possible link of greater cognitive impairment in PD patients with mutations in *GALC* gene was evidenced [97].

Huntington’s disease is a neurodegenerative disease strongly correlated with the expansion of CAG trinucleotide repeat within the *huntingtin* gene (*HTT*). It is characterized by progressive neurodegeneration and cognitive, motor and behavioral disturbances. Different studies carried out in in vivo models of Huntington’s disease have discovered a dysregulation in ganglioside metabolism [98,99]. Furthermore, a recent work has described a downregulation of SPT and CerS in mouse models, with a decrease in dihydroSphingosine, dihydroSphingosine-1-phosphate and dihydroCeramide (C_18_) [100].

Multiple sclerosis, also known as encephalomyelitis disseminate, is a chronic inflammatory disorder of the central nervous system characterized by demyelination and subsequent degeneration leading to neuronal damage and axonal loss. Its underlying etiology is unknown; however, genetic and environmental risks related to its development have been described [101]. CerS2 was found upregulated in monocytes and neutrophils isolated from mouse models [102]; meanwhile, CerS6 was increased in monocytes/macrophages [103,104]. Their overexpression has been associated with an increase in granulocyte stimulating factor (G-CSF)-induced C-X-C Motif Chemokine Receptor 2 (CXCR2) expression [103]. Additionally, downregulation of CerS2 and CerS6 were shown to inhibit the migration capacity of macrophages and neutrophils [103,104]. Therefore, CerS2 and CerS6 may represent a promising target for multiple sclerosis treatment. Moreover, plasma levels of C_16_-Cer, C_24:1_-Cer, C_16_-GlcCer and C_24:1_-GlcCer were increased and C_16_-LacCer was decreased in multiple sclerosis patients compared to healthy controls [105]. Furthermore, increased levels of C_16:0_- and C_24:0_-Cer were found in the cerebrospinal fluid samples from patients with multiple sclerosis [106].

Vascular dysfunction has been associated with the risk of neurodegeneration [107]. Notably, cerebral ischemia has been linked to pro-inflammatory processes in endothelial cells and loss of the integrity of the blood–brain barrier [108,109]. Sphingolipid metabolism has been described as a key factor in the progression and prognosis of brain ischemia. SMS1 was expressed in a time-dependent manner with a decrease in the first 24 h and recuperation at 72 h after transient middle cerebral artery occlusion (tMCAO) in rats [110]. Additionally, mice lacking aSMase exhibited a reduction in the infarct size in tMCAO, related to a decrease in Cer levels [111]. Moreover, a recent study demonstrated that aSMase protects against mild focal cerebral ischemia [112]. In preclinical studies, the levels of ceramides were increased 24 h after tMCAO in the ipsilateral hemisphere, especially in long-chain Cers, and decreased in SM [113]. Furthermore, recent studies in stroke patients showed elevated levels of long-chain Cers, while S1P and very long-chain Cers were decreased. Interestingly, high levels of long-chain Cers were associated with poor outcome at 48–72 h [114,115].

Glioblastoma is the most common and aggressive malignant brain tumor diagnosed in adults. The sphingolipids metabolism has emerged as a potential target for tumor cancer [116]. SPT inhibition by myriocin or specific siRNA inhibited the proliferation of human U87MG glioblastoma cells [117]. Des1 inhibitors such as γ-tocotrienol, phenoxodiol, or celecoxib have been described to induce autophagy in T98G and U87MG glioblastoma cell lines by dhCer accumulation [118]. Furthermore, *N*-[(1R,2S)-2-hydroxy-1-hydroxymethyl-2-(2-tridecyl-1-cyclopropenyl)ethyl]octanamide (GT11), another specific inhibitor of Des1, has been found to activate autophagy and apoptosis of the human U87MG glioma cell line [119]. Additionally, treatment with tetrahydrocannabinol (THC) produced an alteration of the lipid composition in the endoplasmic reticulum and reduction of Des1 expression, promoting autophagy and apoptosis in human U87MG glioma cells [119]. Interestingly, a correlation between SphK1 and poor survival has been observed in a clinical study with patients with glioblastoma [120]. Moreover, specific inhibition of SphK1 or SphK2 resulted in a cell-cycle arrest in U-1242 and U-87MG glioblastoma [120]. In addition, chemical or transcriptional down-regulation of SphK1 induces apoptosis and suppresses the growth of human glioblastoma cells and xenografts [121].

## 4. Ceramide Metabolism Alterations in Parkinson’s Disease

**Figure 2:**
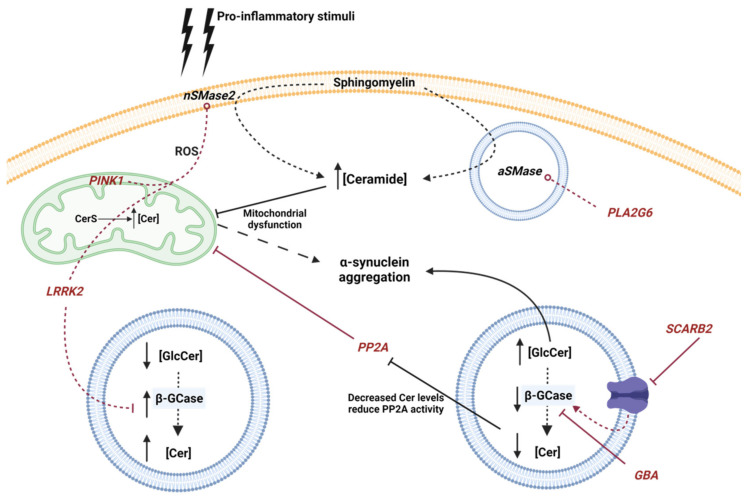
Schematic representation of the influence of genetic mutations involved in PD with Cer metabolism. Genes are represented in italics and red color. Red lines circled at the end indicate increased activity, while a traversed lines at the end indicate inhibition or dysfunction. Neutral Sphingomyelinase 2 (nSMase2), acid sphingomyelinase (aSMase), Ceramide Synthase (CerS), acid β-glucosidase (β-GCase), Ceramide (Cer), GlucosylCeramide (GlcCer) and reactive oxygen species (ROS) are represented by their acronyms.

The causes of PD are not completely understood and vary, from environmental factors such as exposure to toxins, such as rotenone [122] or l-methyl-4-phenyl-1,2,3,6-tetrahydropyridine (MPTP) [123], to genetic factors. About 15 percent of patients with PD have a family history of the condition [3]. Family linked cases can be a consequence of genetic modifications, considered as genetic risk factors, in some of the genes described so far, such as *GBA*, *LRRK2*, *PLA2G6*, *PINK1* or *SNCA* genes (Figure 2).

### 4.1. Genetic Risks

#### 4.1.1. GBA

*GBA* gene mutations are the most common autosomal dominant genetic cause of PD [124]. This gene encodes the lysosomal enzyme β-GCase, a lysosomal hydrolase that degrades GlcCer into Cer and glucose, and alternatively, degrades GlcSph and potentially other β-glucosides. Mutations in this gene that cause loss of expression or functionality in neurons or glia lead to Gaucher’s disease, belonging to Lysosomal Storage Diseases (LSD), by GlcCer accumulation [53,125,126]. Moreover, heterozygous mutations increase the lifetime risk to PD, since increased levels of GlcCer stimulate α-syn pathology [124,127,128]. In addition, recently, it was observed that in a GBA mutant (N370S, L444P, KO) crossed with α-syn transgenic mouse there is a correlation between GlcSph accumulation and α-syn aggregation, indicating that GlcSph promotes α-syn assemblage [129]. Their interconnection is so tight that α-syn has also been shown to reduce β-GCase activity [130]. In fact, elevated levels of GlcCer can lead to α-syn accumulation and, conversely, α-syn can reduce β-GCase activity establishing a bidirectional pathogenic loop [130]. Recently, Jong Kim et al. showed that β-GCase deficiency leads to C_18_-Cer reduction and alters Rab8a location, a small cytosolic GTPase implicated in secretory autophagy [131]. In this work, the authors also demonstrated that ASAH1 inhibition increased C_18_-Cer and reduced GlcSph levels and oxidized α-syn [131]. The latter studies demonstrated the possible efficiency of a targeted therapy against GlcCer accumulation. Sardi et al. demonstrated that the use of a specific GCS inhibitor (GZ667161) in α-synucleinopathy transgenic mice reduced GlcCer and GlcSph levels, correlating with the reduction of α-syn aggregates and cognitive improvement [132].

A new small molecule (S-181) that increases β-GCase activity has recently been developed. In human induced pluripotent stem cell (iPSC)-derived dopaminergic neurons from PD patients carrying mutations in *GBA*, *LRRK2*, *DJ-1* or *PARKIN*, it was found that S-181 reduced β-GCase activity. Furthermore, S-181 partially restored lysosomal function, and consequently, reduced oxidized dopamine, GlcCer levels and α-syn accumulation. Moreover, S-181 treatment of Gba1^D409V/-^ mice resulted in an increased wild-type β-GCase activity and consequent reduction of α-syn accumulation [133]. These results are in agreement with previous studies in which overexpression of β-GCase carried out by Adenovirus Vector (AAV) Gene Therapy observed a reduction of the α-syn aggregation [134,135].

Interestingly, *GBA* deficiency promotes a down-regulation of protein phosphatase 2A (PP2A) leading to an inhibition of autophagy and thus an accumulation of α-syn [136]. This result could be related to a stimulation of PP2A activity by Cer [137,138]. Additionally, it was observed that in lung epithelial cells, the pro-inflammatory cytokine TNF-α stimulates the concentration of intracellular Cer, promoting the stimulation of PP2A activity. Moreover, PP2A was described as modulator of c-Jun N-terminal kinases (JNK), extracellular signal-regulated kinase (ERK) and p38 pathways, stimulating the production of interleukin 8 (IL-8) and along with a pro-inflammatory cascade [139]. Therefore, a low concentration of Cer, due to a deficiency in GBA might produce a decrease in the activity of PP2A. 

It is well established that α-amino-3-hydroxy-5-methyl-4-isoxazolepropionic acid receptors (AMPAR) are the major receptors responsible for synaptic plasticity in neurons. In neurodegenerative diseases, this ability of neurons to adapt fails, leading to cognitive impairments [140]. Among all the proteins that regulate vesicular traffic and AMPAR activation in synapses, PP2A is a relevant enzyme. This phosphatase regulates the dephosphorylation of AMPARs and thus the inactivation and endocytosis of the receptors [141,142]. Furthermore, it has been reported that Cer reduces neuronal AMPAR levels in synapses after a treatment with pro-inflammatory molecules, such as IL-1β [143] or TNF-α [144]. These observations suggest that Cer regulates the activity of AMPARs through PP2A, although these aspects require further investigation. 

Another point of interest is the fact that Cer stimulates autophagy via inhibition of protein kinase B (PKB, also known as Akt) and activation of a mammalian tumor suppressor called Beclin-1 (BENC1), reducing the levels of Cer in GBA deficient neurons, which also produces a decrease in autophagy through this pathway [145]. 

#### 4.1.2. LRRK2

*LRRK2* is a member of the leucine-rich repeat kinase family of genes. Certain mutations associated with PD have been described, representing 1–2% of cases. Mutations in this autosomal dominant gene lead to an increase of *LRRK2* activity [146]. Furthermore, different LRRK2 mutations have been associated with mitochondrial dysfunction, stimulation of reactive oxygen species (ROS) production, dysfunction in fission and fusion processes, mitophagy and dysregulation of cytoskeleton dynamics and mitochondria trafficking [147]. The predominant mutation is the modification of a glycine in the 2019 residue for a serine (LRRK2-G2019S) [148]. Recently, Boecker et al. demonstrated that hyperactivation of LRRK2 kinase activity increases phosphorylation of Rab GTPases and recruits the motor adaptor JNK-interacting protein 4 (JIP4) to the autophagosome membrane, leading to a disruption of autophagosome transport [149]. These observations were made in human iPSC-derived neurons gene-edited to express the G2019S mutation, and the results were reversed by genetic or pharmacological inhibition of LRRK2, making it a hopeful target for PD. 

*LRRK2* mutations have also been observed in patients with *GBA* mutations. However, the phenotype associated with carriers of individual *GBA* or *LRRK2* mutations and the two joint mutations is not clear. A study with a cohort of 236 participants showed that the *LRRK2* mutation together with *GBA* rescued from further mental degeneration [150]. Meanwhile, a recent study with a cohort of 1193 Ashkenazi Jewish population showed no differences between genotypes [151].

Ferrazza et al. demonstrated that *LRRK2* activity is involved in the regulation of Cer metabolism. The authors observed that *Lrrk2^-/-^* transgenic mice showed increased β-GCase activity with elevated Cer levels in the brain [152]. Furthermore, increased β-GCase activity was determined in blood samples from patients with LRRK2-G2019S-PD [127]. This observation could be explained by the release of the lysosomal vesicles to the extracellular compartment [127]. Additionally, recent studies in neurons derived from PD patients with mutations in *LRRK2* have shown that LRRK2 regulates β-GCase activity through the small GTPase Rab10. Moreover, treatment with specific LRRK2 inhibitors (in clinical phase trials) increased β-GCase activity improving cognition functions in PD patients [153]. 

It is well established that LRRK2 regulates mitochondrial homeostasis by Dynamin-related protein (Drp-1) and ROS production [154,155]. In addition, Drp-1 modifies Cer distribution in the outer membrane of the mitochondria, preventing mitophagy [156]. As mentioned above, there is a high expression of mitochondrial CerS in the brain [31]. Interestingly, genetic modifications of CerS in *C. elegans* have been linked to alterations in the inclusion of α-syn [157]. This result could be one of the explanations for the different species of Cer found in patient samples, in both blood and cerebrospinal fluid (CSF), as well as brain biopsies (elegantly reviewed by Pujol-Lereis [22]).

#### 4.1.3. PLA2G6

*PLA2G6* gene encodes a calcium-independent phospholipase A_2_ (iPLA_2_-VIA). This enzyme catalyzes the hydrolysis of glycerophospholipids, producing free fatty acids, including pro-inflammatory polyunsaturated arachidonic acid (AA), and lysophospholipids. Earlier, it was observed that mutations in this gene produce abnormal growth of neuronal axons [158], early onset PD [159,160], and association with PD develop [159]. Additionally, it was also observed that these mutations caused dysregulation of Ca^2+^ homeostasis, leading to autophagic dysfunction and loss of dopaminergic neurons [161]. Interestingly, the loss of iPLA_2_-VIA results in a build-up of Cer [162], by a possible mechanism that alters the plasma composition. In fact, PLA2G6 binds to VPS35 (also mutated in some PD patients [163]) and VPS26, stimulating plasma membrane recycling. This process could stimulate the production of Cer in the lysosomes by aSMase. It should be noted that production of lysosomal Cer leads to α-syn accumulation [87].

Since Cer is the substrate for CerK, C1P concentration can be enhanced by increasing Cer levels. However, C1P levels in the blood of PD patients carrying the *GBA* mutations are reduced in comparison with non-carrier PD patients [164]. Nonetheless, the implication of CerK in PD requires further investigation.

#### 4.1.4. PINK1

The *PINK1* gene encodes for phosphatase and tensin homolog (PTEN)-induced putative kinase protein 1 (PINK1), a mitochondrial-targeted serine/threonine kinase [165]. This protein is implicated in the maintenance of mitochondrial homeostasis. When the mitochondrial membrane suffers a depolarization due to oxidative stress, PINK1 recruits the E3 ubiquitin ligase Parkin protein from the cytosol, initiating a process of mitochondrial degradation called mitophagy [166]. One of the most important consequences is the dysfunction of Na^+^/Ca^2+^ exchange regulation [167]. Mutations in the PINK1 gene are the most common cause of recessive familial PD [165]. Gandhi et al. demonstrated that kinase-dead mutant K219M-PINK1 promotes a dysfunction of Na^+^/Ca^2+^ exchanger with the consequent accumulation of Ca^2+^ in the mitochondria [167]. Calcium overload in the mitochondria stimulates NAPDH oxidase and the production of ROS. The disruption of the electron chain also causes a decrease in glucose transporters in the membrane of neurons by decreasing cellular metabolism. Different studies have shown that the accumulation of ROS stimulates the activity of nSMase2 and the production of Cer [168,169]. Interestingly, the elevation of Cer levels can also take place in the mitochondria by the action of ASAH1 and ASAH2. Moreover, Cer was described to stimulate mitophagy by a mechanism that involves the recruitment of the PINK1/Parkin complex to the mitochondrial membrane [170].

As mentioned previously, PP2A is involved in the regulation of mitochondrial autophagy. In mouse striatum neurons infected with PINK1 gene silencing lentivirus vectors (LVs) an increase in the phosphorylation of PP2A was observed at residue tyrosine 307 (Y307). This phosphorylation resulted in an inactive form of PP2A in dopaminergic cells and striatum tissues, which indicates the involvement of PP2A in the maintenance of mitochondrial homeostasis by PINK1 [171]. Hannun’s group has already shown that the administration of exogenous short-chain Cer (C_2_-Cer) stimulates the activity of PP2A [172]. In the mentioned work, the authors described that the incubation of PINK1-silenced in differentiated dopaminergic MN9D cells with C_2_-Cer activated PP2A and thus reduced autophagy levels in a B-cell lymphoma 2 (Bcl-2)-dependent mechanism, a protein implicated in mitochondrial permeabilization and apoptotic processes [171].

#### 4.1.5. SCARB2

*SCARB2* gene encodes to lysosomal integral membrane protein 2 (LIMP2), a protein responsible for transporting β-GCase from the ER to the lysosome through interaction with mannose-6-phosphate receptor [88,173]. Different genetic studies have determined the implication of mutations in this gene in the development of PD [174,175,176,177,178]. Moreover, overexpression of α-syn in ASO*^Tg/Tg^* mouse models showed reduced levels of mannose-6-phosphate receptor type I (also known as MPR300) [179]. Interestingly, Rothaug et al. demonstrated that the loss of LIMP-2 causes a reduction in β-GCase activity that results in a build-up of α-syn, and thus neurotoxicity in dopaminergic neurons [180]. 

### 4.2. Enviromental Risks

Different neurotoxic agents have been described to affect dopaminergic neurons. In this section, a brief summary of some of these agents will be made, and their relationship with Cer metabolism will be addressed. 

MPTP is a chemical compound able to cross the blood–brain barrier (BBB). Once in the brain, monoamine oxidase B enzyme (MAOB) catalyzes MPTP to produce a 1-methyl-4-phenylpyridinium ion (MPP^+^), a toxic metabolite that accumulates in dopaminergic neurons and inhibits mitochondrial complex I [181]. As a consequence, there is a reduction in ATP levels and generation of free radicals. Furthermore, MPP^+^ increase vesicular dopamine in cytoplasm, where it can also produce ROS [182]. However, this PD model does not present Lewy bodies, one of the characteristic hallmarks of PD. Meanwhile, Rotenone is a lipophilic pesticide found in some tropical plants. Due to its chemical characteristics, it can cross the BBB and internalize in dopaminergic neurons. Like MPP^+^, Rotenone blocks mitochondrial complex I and stimulates ROS production. In addition, Rotenone also inhibits antioxidant enzymes, such as superoxide dismutase (SOD), catalase or glutathione (GSH). This increase in ROS and the inhibition of enzymes involved in their reduction causes degeneration of dopaminergic neurons in the *substantia nigra* with develop of Lewy Bodies. Moreover, it has also been reported that Rotenone induces inflammation, depolarization of microtubules and inhibition of autophagy [183]. 

In the hippocampus of PD mouse models induced by MPTP was observed that nSMase was down-regulated [50]. Previously, 1,25-dihydroxyvitamin D3 was described as activator of SMase activity [184]. In vitro studies in hippocampal PD mice neurons induced by MPTP with consequent upregulation of iNOS and downregulation of nSMase, showed a reduced levels of SM by upregulation of nSMase by 1,25-dihydroxyvitamin D3, related with an improve in synaptic plasticity [50]. Interestingly, treatment with MPP^+^ showed a significant alteration in SphKs and S1P lyase expression in SH-SY5Y dopaminergic neuronal cells [185,186].

Several reports have shown evidence suggesting a critical role of the Toll like receptor 4 (TLR4) in inflammatory responses and neuronal death [187,188,189,190]. Both the microglia- mediated inflammatory pathway and aggregated α-syn activate different mechanisms to stimulate inflammation and contribute to neurodegenerative progression in PD via upregulation of TLR2 and TLR4 [191]. Recent studies in TLR4 knockout mice showed protection against MPTP toxicity, with attenuation of motor deficits and a reduction in α-syn dysfunction and neuroinflammation [187]. Interestingly, another study with these mice described an increase in the expression of nSMase that led to a decrease in SM levels and an increase in Cer, which causes a higher sensitivity to MPTP [192].

Rotenone has been described to inhibit PP2A, preventing the dephosphorylating of α-syn and, in turn increasing its aggregation [193]. Furthermore, C_2_-Cer activates PP2A and counteracted the neurotoxic effects derived from Rotenone treatment and decrease α-syn aggregation [193]. Additionally, in erythrocytes that are known to lack mitochondria, treatment with Rotenone stimulates apoptosis due to an increase in Cer levels, by a mitochondrial-independent mechanism [194]. As showed above, Rotenone inhibits also GSH. Studies in hepatocytes demonstrated that the decrease in GSH activity was correlated with increases in the activity of nSMase2, and this was related to an IL-1β hyperresponsiveness, increasing the pro-inflammatory response [195]. Interestingly, recent study has demonstrated that ASAH1 overexpression increase GSH levels and reduce oxidative stress in fibroblasts derived from Niemann-Pick’s disease type C1 [92].

## 5. Human Sphingolipidomics of Ceramide Metabolism in PD

In recent years, studies on sphingolipid levels by mass spectrometry (sphingolipidomics) have gained importance in different fields of medicine and scientific research [196] (Table 1). It has been reported that sphingolipids metabolism is related to the progression of different carcinogenic processes, as well as resistance to anti-cancer treatments [197,198,199]. Therefore, the study of the evolution of the different species will allow a more effective diagnosis and treatment. Regarding PD, differences in the levels of Cer, SM and GM1, among other sphingolipids, have been described in post-mortem brain samples, blood and CSF from patients with different neuronal degeneration [17,22,200].

PD post-mortem brain tissue from the anterior cingulate cortex showed a drastic reduction in long-chain Cer (such as C_24:1_ and C_24:0_-Cer) levels. Interestingly, an upregulation of CerS1 and CerS4 was observed, which could be due to a compensation system to increase certain Cer species [201]. Meanwhile, comparing plasma samples from patients with *GBA* mutations and non-carrier patients, an increase in Cer and LacCer levels were described, in addition to a decrease in C1P levels [164]. Additionally, in a recent study of post-mortem CSF samples have been described an increase in Cer and SM levels, which correlated with neuropathological staging and disease duration, a fact that further supports the ability of sphingolipids as biomarkers [202]. Moreover, plasma C_14:0_ and C_24:1_-Cer levels were significantly higher in PD with dementia than in PD with no cognitive impairment and normal controls. Interestingly, C_22:0_, C_20:0_ and C_18:0_-Cer were associated with hallucinations, anxiety and disturbances of sleep behavior, respectively [203]. Along with these observations, also plasma Cer (C_16:0_, C_18:0_, C_20:0_, C_22:0_ and C_24:1_) species were found elevated in PD non-*GBA* mutation carriers, with higher levels associated with worse cognitive function [204].

A general increase of long chain SM and Cer levels was also detected in the primary visual cortex [205], as well as, an increase of SM levels in the substantia nigra but only in male PD patients [206]. However, no changes in sphingolipids were observed in the putamen or cerebellum of GBA PD patients [207]. Nonetheless, one study reported reduced plasma levels of SM (d30:1, 32:1 and 39:1) in early PD patients compared to controls [208]. 

Some GlcCer species, including C_18:0_, C _20:0_, C _22:0_, C _24:1_ and C _24:0_ GlcCer in brain tissue, revealed a correlation between increased levels of these metabolites and PD severity [209]. In addition, plasma GlcCer (C_16:0_, C_22:0_ and C_24:0_) species were increased in PD non-*GBA* mutation carriers, and were correlated with greater cognitive impairment [204]. In contrast, GlcCer C_24:1_ was selectively decreased in PD patients with a reduction of C_18:1_-Cer in frontal cortex [210]. Moreover, no significant differences have been found between the different PD groups and controls in CSF samples [211]. 

Gangliosides are complex sphingolipids involved in the regulation of inflammatory processes. Their involvement in PD development has been observed in several studies, although the mechanisms are still uncertain [212]. GM1 can increase the internalization process of α-syn by the glia, increasing α-syn clearance [213] and inhibit the aggregation [214]. By contrast, GM3 accelerates α-syn aggregation by a mechanism dependent on the biophysical conditions of both molecules [215].

Ganglioside GM1 has been reported to be decreased in dopaminergic neurons in substantia nigra of PD patients versus age-matched controls [216]. In plasma from PD patients with *GBA* mutations and/or *LRRK2-G2019S* mutations, increased levels of ganglioside-NANA-3 (a precursor of complex sphingolipids) were observed, possibly due to a reduction in the activity of β-GCase [217]. In addition, in non-carriers of *GBA* or *LRRK2-G2019S* mutations PD patients, GM3 species (d18:1/24:1 and d18:1/26:0) were found elevated in comparation with controls, correlated with high levels of GlcCer species in plasma [218].

**Table 1:** Summary table of human sphingolipidomics. The up arrows refer to an increase in levels compared to the controls, while the down arrows refer to a reduction. The asterisk (*) indicates that the data were not statistically significant. Ceramide (Cer), ceramide 1-phosphate (C1P), sphingomyelin (SM), glucosyl-ceramide (GlcCer) and lactosyl-ceramide (LacCer).

Sphingolipid Species	Levels	Source	Type of PD	Notes	Ref.
Cer	↑	Serum	*GBA* mutation	Possible early development of PD and worsening of symptoms	[164]
	↑	*Post-mortem* CSF	Not specified	Correlation with neuropathological staging and disease duration	[202]
	↑	Primary visual cortex	Sporadic	Contribution to neuronal dysfunction	[205]
Long-chain Cer (such as C_24:1_ and C_24:0_-Cer)	↓	Anterior cingulate cortex		Impartment of salvage pathway by increased CerS1 expression	[201]
C_14:0_-Cer	↑	Plasma	PD with dementia	Association with delayed free recall and cognition	[203]
C_16:0_-Cer	↑	Plasma	Non-*GBA* mutation	Association with worse cognition	[204]
C_18:0_-Cer	↑	Plasma	Non-*GBA* mutation	Association with worse cognition	[204]
	↑ *		PD with dementia	Association with sleep behaviour disturbance	[203]
C_18:1_-Cer	↓	Frontal cortex	Not specified	Increased formation of diacylglycerols (DAGs)	[210]
C_20:0_-Cer	↑ *	Plasma	PD with dementia	Association with anxiety	[203]
	↑		Non-*GBA* mutation	Association with worse cognition	[204]
C_22:0_-Cer	↑	Plasma	Non-*GBA* mutation	Association with worse cognition	[204]
	↑ *		PD with dementia	Association with hallucination	[203]
C_24:1_-Cer	↑	Plasma	PD with dementia	Association with the score of immediate verbal recall and delayed free recall	[203]
	↑		Non-*GBA* mutation	Association with worse cognition	[204]
GlcCer	↑	Plasma	Non-GBA or Non-LRRK2 G2019S mutation	Association with worse cognition	[204,218]
GlcCer C_16:0_	↑	Plasma	Non-*GBA* mutation	Association with worse cognition	[204]
GlcCer C_18:0_	↑ *	Temporal cortex	Not specified	Correlation with PD severity	[209]
GlcCer C_20:0_	↑ *	Temporal cortex	Not specified	Correlation with PD severity	[209]
GlcCer C_22:0_	↑ *	Temporal cortex	Not specified	Correlation with PD severity	[209]
	↑	Plasma	Non-*GBA* mutation	Tendency to association with worse cognition	[204]
GlcCer C_24:0_	↑	Plasma	Non-*GBA* mutation	Association with worse cognition	[204]
	↑ *	Temporal cortex	Not specified	Correlation with PD severity	[209]
GlcCer C_24:1_	↑ *	Temporal cortex	Not specified	Correlation with PD severity	[209]
	↓	Frontal cortex	Not specified	Increased formation of DAGs	[210]
LacCer	↑	Serum	*GBA* mutation	Proposed as novel biomarker for increased risk of PD develop	[164]
C1P	↓	Serum	*GBA* mutation	Proposed as novel biomarker for increased risk of PD develop	[164]
SM	↑	*Post-mortem* CSF	Not specified	Correlation with neuropathological staging and disease duration	[205]
	↑	*Substantia nigra*	Male PD	Caused by enrichment in Lewy bodies	[206]
Long-chain SM	↑	Primary visual cortex	Sporadic	Contribution to neuronal dysfunction	[205]
SM d30:1	↓ *	Plasma	Not specified	Due to dysregulation of sphingolipids metabolism. Possibly involved in demyelination	[208]
SM d32:1	↓ *	Plasma	Not specified	Due to dysregulation of sphingolipids metabolism. Possibly involved in demyelination	[208]
SM d39:1	↓ *	Plasma	Not specified	Due to dysregulation of sphingolipids metabolism. Possibly involved in demyelination	[208]
GM1	↓	*Substantia nigra* dopaminergic neurons	Sporadic	Loss of neuroprotection and acceleration of α-syn formation	[216]
Ganglioside-NANA-3	↑	Plasma	*GBA* or/and *LRRK2* G2019S mutation	Acceleration of α-syn formation	[217]
GM3 d18:1/24:1	↑	Plasma	Non-*GBA* or Non-*LRRK2* G2019S mutation	Acceleration of α-syn formation	[218]
GM3 d18:1/26:0	↑	Plasma	Non-*GBA* or Non-*LRRK2* G2019S mutation	Acceleration of α-syn formation	[218]

## 6. Concluding Remarks

The dual function of sphingolipids as structural molecules and second messengers makes them vitally important molecules for the maintenance of cell and tissue homeostasis. It has been demonstrated that alterations of sphingolipid metabolism are related to PD progression, and its aggressiveness by a large number of studies carried out in different animal models, in vitro studies and human samples. However, the molecular mechanisms that give rise to this alteration remain incompletely understood. Studies in which the therapeutic targets are the enzymes involved in the metabolism of sphingolipids are of vital importance.

In recent years, sphingolipidomic studies in patient samples have emerged as a new tool to evaluate the aggressiveness and establish specific treatments of certain diseases, including PD. Moreover, sphingolipid levels in PD patients with different symptoms and progress have demonstrated the viability of sphingolipidomics for both diagnosis and specific treatments. These data support the potential of sphingolipids as biomarkers of PD. Furthermore, transcriptional studies have shown variations in the expression of different enzymes involved in sphingolipid metabolism. However, only a few studies have been conducted to determine their involvement in neurological impairment in PD.

Future experiments that aim to understand the involvement of sphingolipids in the development of PD will be of vital importance for the development of therapies and an early diagnosis.
